# Visual cortex anodal transcranial direct current stimulation does not alter reading performance for Chinese presented character-by-character to normal peripheral vision in older adults

**DOI:** 10.3389/fnins.2024.1341307

**Published:** 2024-04-24

**Authors:** Anqi Lyu, Andrew E. Silva, Benjamin Thompson, Larry Abel, Allen M. Y. Cheong

**Affiliations:** ^1^School of Optometry, The Hong Kong Polytechnic University, Kowloon, Hong Kong SAR, China; ^2^School of Optometry and Vision Science, University of Waterloo, Waterloo, ON, Canada; ^3^Centre for Eye and Vision Research Limited, Hong Kong, Hong Kong SAR, China; ^4^School of Medicine, Deakin University, Burwood, VIC, Australia; ^5^Research Centre for SHARP Vision, The Hong Kong Polytechnic University, Kowloon, Hong Kong SAR, China

**Keywords:** transcranial direct current stimulation, tDCS, Chinese reading, peripheral vision, RSVP

## Abstract

Visual cortex anodal transcranial direct current stimulation (a-tDCS) has been shown to reduce crowding in normal peripheral vision and may improve the reading of English words in patients with macular degeneration. Given the different visual requirements of reading English words and Chinese characters, the effect of a-tDCS on peripheral reading performance in English might differ from Chinese. This study recruited 20 participants (59–73 years of age) with normal vision and tested the hypothesis that a-tDCS would improve the reading of Chinese characters presented at 10° eccentricity compared with sham stimulation. Chinese sentences of different print sizes and exposure durations were presented one character at a time, 10° below or to the left of fixation. The individual critical print size (CPS) – the smallest print size eliciting the maximum reading speed (MRS) – was determined. Reading accuracies for characters presented 0.2 logMAR smaller than the individually fitted CPS were measured at four time points: before, during, 5 min after, and 30 min after receiving active or sham visual cortex a-tDCS. Participants completed both the active and sham sessions in a random order following a double-blind, within-subject design. No effect of active a-tDCS on reading accuracy was observed, implying that a single session of a-tDCS did not improve Chinese character reading in normal peripheral vision. This may suggest that a-tDCS does not significantly reduce the crowding elicited within a single Chinese character. However, the effect of a-tDCS on between-character crowding is yet to be determined.

## Introduction

Reading speed is slower when using peripheral vision compared with central vision (Chung et al., 1998; Chung, 2002; Yu et al., 2006), possibly due to reduced spatial resolution (Masland, 2017), poorer eye movement control (Whittaker et al., 1991; Bethlehem et al., 2014), restricted visual span (Legge et al., 2001), and susceptibility to crowding (Chung, 2002, 2004). Although few studies have investigated the factors affecting Chinese reading performance in peripheral vision (Zhang et al., 2009), our recent findings reveal systematic impairments in peripheral Chinese reading, including slower temporal processing speeds and reduced spatial visual spans (Lyu et al., 2022). Understanding the peripheral reading performance in readers with normal vision may help generate better rehabilitation solutions for the low-vision community.

Clinically, reading difficulty is a major concern for patients with age-related macular degeneration (AMD), who are compelled to rely on para-central or peripheral vision due to the presence of a central scotoma. Studies of interventions designed to improve normal peripheral vision not only enhance our understanding of the effects of interventions on reading performance but may also facilitate their future adoption for patients with AMD. For example, increasing English letter spacing (within a particular range) to reduce crowding improved peripheral reading speed (Latham and Whitaker, 1996; Chung, 2002). Multiple training sessions on a crowded letter identification task also improved peripheral English reading performance (Chung, 2007).

Recently, non-invasive brain stimulation (NIBS), which enables the modulation of neural activity in targeted superficial areas of the human brain, is emerging as a promising tool for vision enhancement and rehabilitation. Anodal transcranial direct current stimulation (a-tDCS), a common NIBS technique (Nitsche and Paulus, 2000), has been demonstrated to improve a range of visual functions. Results from a recent meta-analysis illustrated that visual cortex a-tDCS improved contrast sensitivity, visual evoked potential amplitude (an index of cortical excitability), and crowding in peripheral vision among normally sighted individuals (Bello et al., 2023). TDCS involves a weak 1 – 2 mA electrical current delivered through two head-mounted electrodes (the anode and cathode) and induces regional changes in cortical excitability and neurotransmitter concentrations that outlast the duration of stimulation [see Roche et al. (2015) for a review]. Several studies have explored the effect of visual cortex a-tDCS on normal peripheral vision. Reinhart et al. (2016) showed that a single 20-min session of a-tDCS applied to the visual cortex significantly improved vernier acuity at 5° eccentricity in 20 normally sighted subjects. Furthermore, Raveendran et al. (2020) examined the acute effect of a-tDCS on collinear lateral inhibition, which is one of the low-level inhibitory mechanisms contributing to visual crowding, in 13 subjects with normal vision. The participants detected a central target presented 6° left of fixation which was vertically flanked by two colinear Gabor patches. Lateral inhibition was significantly reduced following 20 min of visual cortex a-tDCS whereas sham stimulation had no effect (Raveendran et al., 2020). Raveendran et al. (2020) proposed that the early stages of visual processing of peripheral stimuli could be enhanced by a-tDCS, potentially benefiting the high-level visual processing of crowded stimuli. Indeed, Chen et al. (2021) demonstrated that the contrast threshold for recognizing an English letter at 10° eccentricity crowded by two random letters decreased (an improvement of 23%) after 20 min of active a-tDCS, but not sham stimulation.

The reduction of crowding in peripheral vision resulting from visual cortex a-tDCS suggests that this intervention may improve peripheral reading. In the first study to address this question, Silva et al. (2022) conducted a randomized, within-subjects experiment to examine the acute effects of visual cortex a-tDCS on English and Chinese reading performance in patients with bilateral central vision loss. They found that 20 min of a-tDCS tended to improve reading accuracy for English compared with sham stimulation. However, reading accuracy for Chinese did not change with a-tDCS (Silva et al., 2022). The authors concluded that the effects of visual cortex a-tDCS on peripheral reading varied for different writing systems. However, reading abilities vary widely in AMD patients depending on their remaining vision and the location of their preferred retinal locus (Fletcher et al., 1999; Altinbay et al., 2016; Farzaneh et al., 2021). It has also been argued that AMD is accompanied by negative peripheral retinal manifestations because of the increased prevalence of peripheral autofluorescence abnormalities [see Pivovar and Oellers (2021) for a review]. Hence, the remaining peripheral visual function might vary substantially across patients. Both of these factors might mask an effect of a-tDCS on reading in patients with AMD. Furthermore, in Silva et al.’s (2022) study, English reading performance was evaluated through a word-by-word presentation of sentences, whereas Chinese reading was examined by presenting sentences using a character-by-character approach. Chinese, as an orthographic language, is characterized by logographic characters exhibiting varying spatial complexities. Chinese reading involves both internal (within-character) and external (between-character) crowding (Zhang et al., 2009). It remains unclear if the finding of a null effect of a-tDCS on Chinese reading was due to the wide variation in reading performance that AMD patients may have, or if it was because a-tDCS did not reduce within-character crowding.

In summary, a-tDCS of the visual cortex can reduce crowding in peripheral vision (Spiegel et al., 2012; Raveendran et al., 2020; Bello et al., 2023), and, on this basis, we explored whether a-tDCS could improve peripheral reading in patients with AMD (Silva et al., 2022). A differential effect for English and Chinese writing systems was found whereby there was no improvement for Chinese readers. Two possible explanations for this null result are: (1) a-tDCS does not reduce the internal (within-character) crowding that occurs in Chinese characters, and (2) the variability in reading impairments caused by the heterogeneous sample of participants with AMD masked an effect. Based on the previous literature indicating a robust effect of a-tDCS on crowding, in this study we tested the hypothesis that active a-tDCS would improve peripheral reading of Chinese characters relative to sham a-tDCS in healthy older adults whose peripheral reading performance is not affected by the disease. We tested reading performance before and after a-tDCS using the same method described in Silva et al. (2022) with individual Chinese characters presented at fixed locations 10° below and 10° left of fixation.

## Materials and methods

### Subjects

Twenty healthy older subjects (aged between 59 and 73 years) with normal or corrected-to-normal vision were recruited from community centers and social media. All subjects spoke and read traditional Chinese as their first language. Eligibility criteria included no history of any ophthalmic, neurological, or psychiatric illness, no taking medications that could affect reading performance, or any contraindication to NIBS such as epileptic seizure or metal/electronic implants in the head. Participants were divided into two groups: (1) reading at 10° left visual field (*n* = 10), and (2) reading at 10° inferior visual field (*n* = 10). The rationale for adopting two peripheral locations was because these are the two most frequent para-central locations adopted by AMD patients fixating at a central cross (33.7% and 39%, respectively) (Fletcher and Schuchard, 1997). Demographic data including age and gender were collected and summarized in Table 1. The best-corrected distance visual acuity was measured with an Early Treatment of Diabetic Retinopathy Study (ETDRS) chart of high-contrast letters at 4 m. The best-corrected near visual acuity was measured using the near ETDRS modified Snellen chart at 40 cm. The Montreal Cognitive Assessment (MoCA) was used to ensure that participants did not have impaired cognition. The average age of the participants reading at 10° left vision (8 females and 2 males) was 65.73 ± 4.69 years, while for those reading at 10° lower vision (5 females and 5 males), it was 65.18 ± 4.19 years. Both groups did not show significant differences in terms of age, gender, visual acuity, or reading speed (Table 1). This experiment was approved by The Department Research Committee of the School of Optometry of The Hong Kong Polytechnic University (HSEARS20190716005). All subjects gave written informed consent. The study followed the tenets of the Declaration of Helsinki.

**TABLE 1:** Demographic data of the subjects.

	10° left visual field (*n* = 10)	10° inferior visual field (*n* = 10)	*p*-Value
Age (years)	65.73 ± 4.69 (59–73)	65.18 ± 4.19 (60–71)	*t*(18) = 0.27; *p* = 0.79
Gender	F: 80% M: 20%	F: 50% M: 50%	*U* = 35; *p* = 0.35
Best-corrected visual acuity (logMAR)	Distance: 0.06 ± 0.06 Near: 0.13 ± 0.09	Distance: 0.02 ± 0.09 Near: 0.07 ± 0.07	*t*(18) = 1.10; *p* = 0.29 *U* = 28; *p* = 0.10
RSVP critical print size (logMAR)*	1.51 ± 0.09	1.65 ± 0.08	*U* = 11; *p* < 0.01
RSVP reading speed (number of characters per minute)*	212.00 ± 51.31	198.06 ± 94.03	*t*(18) = 0.41; *p* = 0.69

LogMAR, logarithm of the minimum angle of resolution measured using the Early Treatment Diabetic Retinopathy Study (ETDRS) chart at 4 m (distance) and 40 cm (near). *The values represent the reading speed and print size that yield 55% accuracy in the RSVP measurement. Two-tailed Student’s *t*-test or Mann–Whitney U test was used to compare the differences between groups. Mean ± SD was calculated across groups.

### Experimental design

**FIGURE 1:**
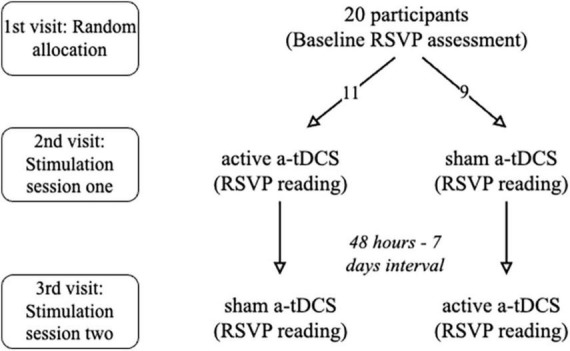
Flow of the study.

A within-subject, double-blind, cross-over design was employed. Subjects were invited to The Hong Kong Polytechnic University Optometry Research Clinic and completed evaluations on three occasions (see Figure 1 for the flow of the study). All participants underwent an initial reading evaluation and the reading performance before and after receiving a-tDCS was compared for both stimulation sessions. All tests were performed using their right eye with full refractive correction and appropriate near addition while the left eye was occluded with an eye patch.

### Visit I – baseline reading assessment

**FIGURE 2:**
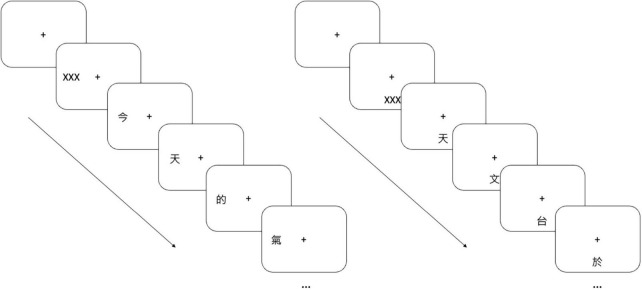
Rapid serial visual presentation (RSVP). Character-by-character sentences were presented 10° left **(left panel)** or 10° below **(right panel)** the fixation cross. Subjects verbally reported the characters they recognized.

Reading performance was assessed using rapid serial visual presentation (RSVP) which involved character-by-character presentation of sentences on a screen (Legge et al., 2001; Chung, 2002, 2011, 2021). We adopted the same approach detailed in Silva et al. (2022). Chinese characters were generated using PsychoPy 2020.1.3 (Peirce et al., 2022) and presented on a 24-inch LCD monitor (BENQ xl2540, 120 Hz refresh rate, 1,920 × 1,080 resolution). Subjects were seated 65 cm in front of the monitor with a chinrest and forehead bar stabilizing their head position. Subjects were asked to maintain fixation on a central cross. First, a short mask of “XXX” was presented at either 10° left of fixation or below fixation. Then, a randomly selected sentence replaced the mask and was presented one character at a time at the same location and with the same size as the initial mask (Figure 2). The sentence was selected randomly from a pool of 605 Chinese sentences comprising 15 characters. Subjects verbally reported all recognized characters in a self-generated and self-timed way, either after all characters disappeared or while the characters were being displayed. There was no repetition of individual sentences among the participants. Eye movements were monitored using an infrared video eye-tracking system (Eyelink Portable Duo, SR Research, Scarborough, ON, Canada). Trials were replaced if fixation deviated by more than 1°. Less than 20% of the trials were replaced for all participants.

**FIGURE 3:**
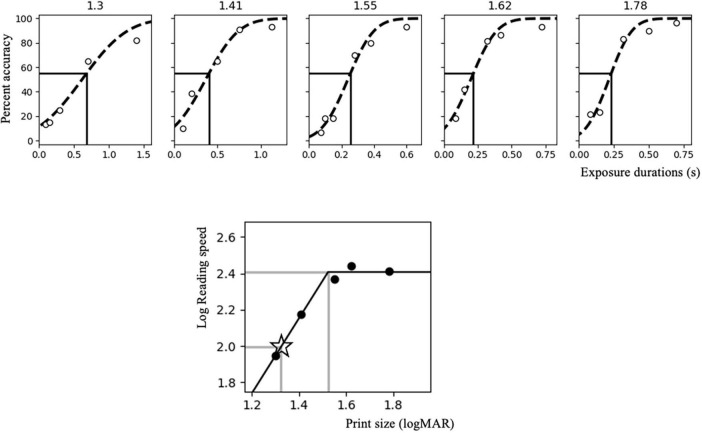
Rapid serial visual presentation (RSVP) curve fitting for a sample subject. The **upper panel** shows a representative RSVP measurement involving five print sizes (1.3, 1.41, 1.55, 1.62, and 1.78 logMAR). The accuracy for each exposure duration is represented by the white circles. The black dotted curve represents the psychometric function (cumulative Gaussian distribution), while the vertical dark line points to the exposure duration that elicits 55% character recognition accuracy. The **lower panel** shows the bilinear function relating log reading speed (dark circles) to a range of tested print sizes. The upper horizontal and vertical gray lines indicate the maximum reading speed (MRS) and critical print size (CPS) corresponding to 55% accuracy. The lower gray lines indicate the reading speed (in terms of exposure duration) and print size used during the stimulation sessions, which corresponds to a size of 0.2 logMAR smaller than CPS (marked by the star).

On the initial visit, participants performed a set of RSVP measurements. Each subject was tested with five print sizes (defined by the vertical height of a square character configuration, ranging from 1.30 to 1.78 logMAR). Each print size was tested using five different exposure durations (i.e., the duration of each sequentially presented character). Additional exposure durations were added in units of 1.5 times the preceding duration until a spread of recognition accuracies between 20% and 80% were collected. Five trials of each exposure duration were presented for each print size, resulting in a total of at least 125 individual sentences per participant. A psychometric function (cumulative Gaussian distribution) relating exposure duration to character recognition accuracy was fitted (see Supplementary Results for individual-subject plots of all fitted psychometric functions). The exposure duration that elicited 55% recognition accuracy for each print size – converted to log characters per minute (log cpm = log (60 / exposure duration (seconds))) – was derived (Silva et al., 2022). Log print size (minimum angle of resolution, logMAR) and log characters per minute were then fitted to a continuous bilinear function separately for each participant. Reading speed was assumed to increase linearly with increasing print size in this bilinear function until reaching a plateau. The reading speed associated with the plateau determined the participant’s maximum reading speed (MRS), and the smallest print size eliciting the MRS determined the critical print size (CPS) (Chung, 2002, 2021; Cheong et al., 2007). The print size corresponding to 0.2 logMAR below the individually fitted CPS and the associated exposure duration were tested in both stimulation sessions (Silva et al., 2022), to provide room for any improvement in reading accuracy (see Figure 3 for an example).

### Visits II and III – anodal transcranial direct current stimulation

**FIGURE 4:**
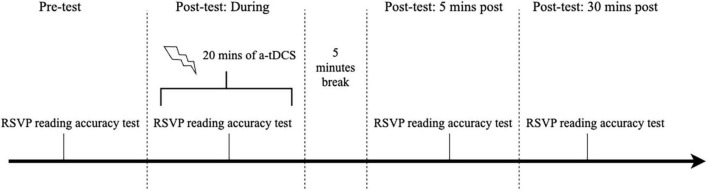
Flow of the testing procedure for each stimulation day.

The a-tDCS sessions comprised two visits separated by at least 48 h but no more than 7 days. A-tDCS was delivered using a battery-powered current stimulator (Neuro Device Group S.A., nurostym tES) and two 5 cm × 5 cm rubber electrodes placed inside saline-soaked sponges. Subjects received either active or sham a-tDCS in random order during the two visits. Both experimenter and subject were blinded to the stimulation type. The anodal electrode was placed over the primary visual cortex (Oz in the international 10–20 electroencephalogram system), and the cathode was randomly applied over the left or right cheek (Silva et al., 2022), secured using a cap. Active a-tDCS involved a direct current ramping up to 2 mA over 30 s, continuously delivered for 20 min, then ramped down to 0 in 30 s. Sham a-tDCS only consisted of the 30-s ramp-up and down periods and no current applied otherwise. While participants were not asked to identify whether they received active or sham a-tDCS, they reported any side effects experienced during each session (see Supplementary Results). During each a-tDCS session, subjects first read 15 RSVP sentences at the assigned peripheral location with the selected print size and exposure duration before receiving stimulation. Subjects then underwent 20 min of a-tDCS and performed an identical reading test with 15 new sentences. The reading task was again administered 5 and 30 min after the cessation of the stimulation (see Figure 4 for the stimulation protocol). The outcome measures were the reading accuracies assessed at the pre-test and the three post-tests within the same stimulation session.

### Statistical analysis

Statistical analysis was performed using GraphPad Prism 9.2.0 for Windows 64-bit, GraphPad Software, San Diego, CA, USA.^1^ RSVP reading accuracies were not significantly different from a normal distribution (Kolmogorov–Smirnov goodness of fit test, *p* > 0.5). The two groups (reading at 10° left vs. inferior visual field) were compared using the two-tailed Student’s *t*-test or Mann–Whitney U test (if data were non-normally distributed) in relation to their age, gender, visual acuity, CPS, and reading speed. RSVP reading accuracies were compared with a repeated-measure analysis of variance (ANOVA) with stimulation type (active vs. sham) and time (pre- vs. during- vs. 5 min post- vs. 30 min post-stimulation) as within-subject factors and stimulus location (left vs. inferior) as a between-subject factor. A *p*-value of less than 0.05 was considered statistically significant.

## Results

**FIGURE 5:**
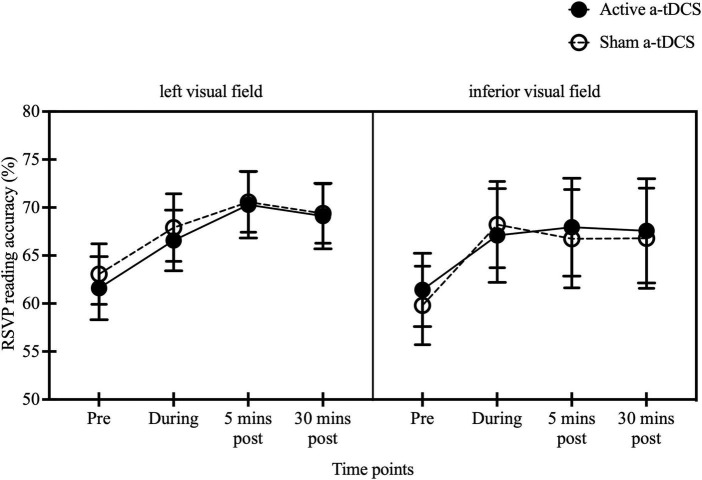
Effect of a-tDCS on RSVP reading accuracies for Chinese characters presented to 10° left and inferior visual fields. Reading accuracies at pre-, during-, and 5 min post- and 30 min post-stimulation at 10° left visual field **(left panel)** and 10° inferior visual field **(right panel)**. The black solid and open circles represent the mean reading accuracies for active and sham a-tDCS respectively. The error bars are ±1 SEM.

Reading accuracy was compared at different time points (pre-stimulation, during stimulation, 5 min post-stimulation, and 30 min post-stimulation) between active and sham a-tDCS, as well as between left and inferior visual fields. A significant effect of time was observed [*F*(3, 54) = 9.12, *p* < 0.001, η^2^ = 0.34]. However, no significant effect of stimulation type [*F*(1, 18) = 0.01, *p* = 0.91, η^2^ = 0.001] or stimulus location [*F*(1, 18) = 0.09, *p* = 0.77, η^2^ = 0.005] was found. The interaction effects between time and stimulation type [*F*(3, 54) = 0.75, *p* = 0.53, η^2^ = 0.04], time and stimulus location [*F*(3, 54) = 0.42, *p* = 0.74, η^2^ = 0.02], stimulation type and stimulus location [*F*(1, 18) = 0.61, *p* = 0.44, η^2^ = 0.03], or among time, stimulation type and stimulus location [*F*(3, 54) = 0.47, *p* = 0.71. η^2^ = 0.03] were not significant. Both groups showed higher reading accuracy during (66.57 ± 3.16 and 67.91 ± 3.51, and 67.09 ± 4.89 and 68.22 ± 4.49 for active and sham a-tDCS in left and inferior stimulus groups, respectively), 5 min post (70.29 ± 3.47 and 70.58 ± 3.15, and 67.96 ± 5.10 and 66.76 ± 5.12) and 30 min post-stimulation (69.11 ± 3.41 and 69.38 ± 3.09, and 67.58 ± 5.43 and 66.80 ± 5.21) compared with pre-stimulation (61.60 ± 3.28 and 63.07 ± 3.15, and 61.42 ± 3.82 and 59.80 ± 4.09, *p* < 0.003). No difference was found between during stimulation and the two post-measures (*p* > 0.99) (Figure 5).

**FIGURE 6:**
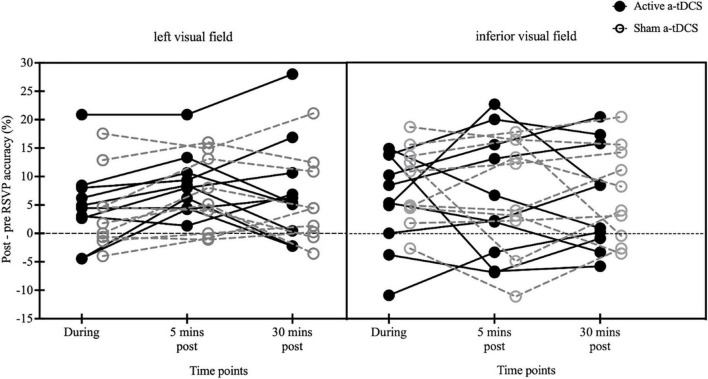
Individual-subject effects of a-tDCS on RSVP accuracy changes for Chinese characters presented to 10° left and inferior visual fields (black solid circles: active; gray open circles: sham). Percent differences in RSVP recognition accuracy at each of the three post-tests (during-, 5 min post-, and 30 min post-stimulation) relative to pre-test at 10° left visual field **(left panel)** and 10° inferior visual field **(right panel)**.

To control for the large inter-subjects variabilities of pre-stimulation reading measures, the changes in reading accuracy were compared among the three post-stimulation tests relative to the pre-stimulation performance (Figure 6). No significant effect of stimulation type [*F*(1, 18) = 0.04, *p* = 0.84, η^2^ = 0.002], time [*F*(2, 36) = 0.48, *p* = 0.62, η^2^ = 0.03], or stimulus location [*F*(1, 18) = 0.002, *p* = 0.96, η^2^ < 0.001] was found. Additionally, the interaction effects between time and stimulation type [*F*(2, 36) = 1.38, *p* = 0.26, η^2^ = 0.07], time and stimulus location [*F*(2, 36) = 0.74, *p* = 0.48, η^2^ = 0.04], stimulation type and stimulus location [*F*(1, 18) = 0.80, *p* = 0.38, η^2^ = 0.04], or among time, stimulation type and stimulus location [*F*(2, 36) = 0.17, *p* = 0.84, η^2^ = 0.01] were not significant (4.97 ± 2.27 vs. 8.69 ± 1.74 vs. 7.51 ± 2.92 and 4.85 ± 2.29 vs. 7.51 ± 2.23 vs. 6.31 ± 2.43 in the left stimulus group and 5.67 ± 2.67 vs. 6.53 ± 3.43 vs. 6.16 ± 2.94 and 8.42 ± 2.14 vs. 6.96 ± 3.12 vs. 7.00 ± 2.60 in the inferior stimulus group for the active and sham a-tDCS, respectively).

## Discussion

The current study aimed to investigate the effect of 20 min of visual cortex active a-tDCS compared with sham a-tDCS on reading individual Chinese characters in a population with normal peripheral vision. In opposition to our hypothesis, we did not find any significant improvement in Chinese reading at peripheral vision following active a-tDCS relative to sham stimulation.

Two peripheral locations were examined in the current study as they represent the two most frequent para-central regions adopted by AMD patients fixating at a central cross (Fletcher and Schuchard, 1997). We did not find a significant difference in accuracy when reading individual Chinese characters presented in these two locations.

Chinese, as a logographic orthography language, contains an enormous number of characters [approximately 2,500 frequently used Chinese characters (Zhu et al., 2019)] with a wide range of spatial complexities arranged in a square configuration. This unique feature makes Chinese reading in the peripheral vision challenging due to two levels of crowding: internal (within-character) and external (between-character) (Zhang et al., 2009). In the current study, Chinese reading performance was examined using a character-by-character presentation which isolated internal crowding. This is in contrast to studies of English reading in peripheral vision which mainly isolate external crowding between adjacent letters (Chung et al., 1998; Yu et al., 2006; Silva et al., 2022). Silva et al. (2022) found that active a-tDCS tended to increase reading accuracy in patients with macular degeneration who read English sentences presented word-by-word compared with sham stimulation, whereas such improvement was not shown in patients reading Chinese character-by-character. Furthermore, Chen et al. (2021) compared the contrast threshold for recognizing either isolated English letters or letters flanked by two distractors presented at 10° eccentricity before and after a-tDCS in normally sighted young subjects. They found that the contrast threshold for recognizing isolated letters was not altered after receiving active a-tDCS. However, a significant improvement was found after the stimulation in the crowded conditions (Chen et al., 2021). These studies suggest that a-tDCS may have a greater impact on alleviating between-character crowding than within-character crowding, thus enhancing character recognition and peripheral reading performance. To further explore the effects of a-tDCS on Chinese reading, future studies should consider presenting multiple Chinese characters simultaneously [e.g., recognizing the central character within a trigram (Lyu et al., 2022)] to investigate the effect of a-tDCS on internal vs. external crowding in peripheral vision. In addition, only a single session of a-tDCS was adopted in the current study. Exploring the effects of multiple sessions of a-tDCS on improving Chinese reading performance is warranted.

Chinese characters represent morphemic syllables that carry contextual information (Liu and Perfetti, 2003). It has been proposed that Chinese characters are predominantly memorized through verbal rather than visual means (Hue and Erickson, 1988). Xu et al. (2015) conducted a functional magnetic resonance imaging study to examine the brain activity of 18 normally sighted young adults while they were performing a word recognition task and found that the ventral pathway which is responsible for visual recognition, instead of the dorsal pathway (involved in spatial processing), plays a critical role in Chinese character processing (Xu et al., 2015). Their results contradicted previous studies conducted in English, which suggested that both the ventral and dorsal pathways connecting the visual cortex to high-level language areas were engaged in processing alphabetic languages (Booth et al., 2008; Levy et al., 2009). Therefore, although both English words and Chinese characters need to be projected to the primary visual cortex for high-level processing, the recognition of Chinese characters involves orthographic processing and the underlying difference in neural pathways may account for the null a-tDCS effect in the current study, compared with English reading. In addition to the primary visual cortex, the visual word form area (VWFA) is involved in letter recognition (Dehaene and Cohen, 2011) and reading performance (Hillis et al., 2005; Chen et al., 2019). A recent study by Chen et al. (2021) has demonstrated that a-tDCS applied to the occipitoparietal region significantly improved the perceiving of English letters in peripheral vision. However, its effect on recognizing Chinese characters and reading is still unclear. Future studies are warranted to determine a more advantageous stimulation location to enhance the effect of NIBS on Chinese reading.

Recent studies have revealed that another form of NIBS – transcranial random noise stimulation (tRNS) which involves an alternating current that randomly changes in frequency and amplitude, may have a larger effect than a-tDCS on cortical activity (Inukai et al., 2016) and visual perception (Fertonani et al., 2011). Enhancement of signal-to-noise ratio within the visual cortex due to stochastic resonance has been proposed as the potential mechanism to explain the enhancing effect of tRNS on vision. It is possible that tRNS may improve the recognition and reading of Chinese characters presented to peripheral vision. However, this speculation requires further study.

## Conclusion

In agreement with our previous work in a group of patients with central vision loss (Silva et al., 2022), active a-tDCS applied to the primary visual cortex did not significantly change Chinese reading performance in normal peripheral vision. Although the relatively small sample size of this study may limit the generalizability of the results, this exploratory study provides insights into the field of NIBS and its association with reading. Because real-world Chinese reading involves multiple characters presented together, future work should use reading materials that present sentences or text to explore the impact of NIBS on replicating real-world reading experiences.

## Data availability statement

The datasets presented in this study can be found in online repositories. The names of the repository/repositories and accession number(s) can be found below: https://figshare.com/articles/dataset/Effect_of_visual_cortex_anodal_tDCS_on_Chinese _reading_performance_in_normal_peripheral_vision_/23306198.

## Ethics statement

The studies involving humans were approved by the Department Research Committee of the School of Optometry of The Hong Kong Polytechnic University. The studies were conducted in accordance with the local legislation and institutional requirements. The participants provided their written informed consent to participate in this study.

## Author contributions

AL: Formal analysis, Methodology, Project administration, Writing – original draft. AS: Methodology, Software, Writing – review & editing. BT: Funding acquisition, Methodology, Writing – review & editing, Supervision. LA: Methodology, Supervision, Writing – review & editing. AC: Funding acquisition, Methodology, Supervision, Writing – review & editing.

## Footnotes

1
www.graphpad.com
