# Supplementary material for: Visual cortex anodal transcranial direct current stimulation does not alter reading performance for Chinese presented character-by-character to normal peripheral vision in older adults
Source: Front Neurosci. 2024 Apr 24;18:1341307. doi: 10.3389/fnins.2024.1341307 (PMC11076872; doi:10.3389/fnins.2024.1341307)
Supplement: Supplementary file 1 [file Data_Sheet_1.PDF]

Supplementary Results

1. RSVP psychometric fitting for each subject

Sub 001

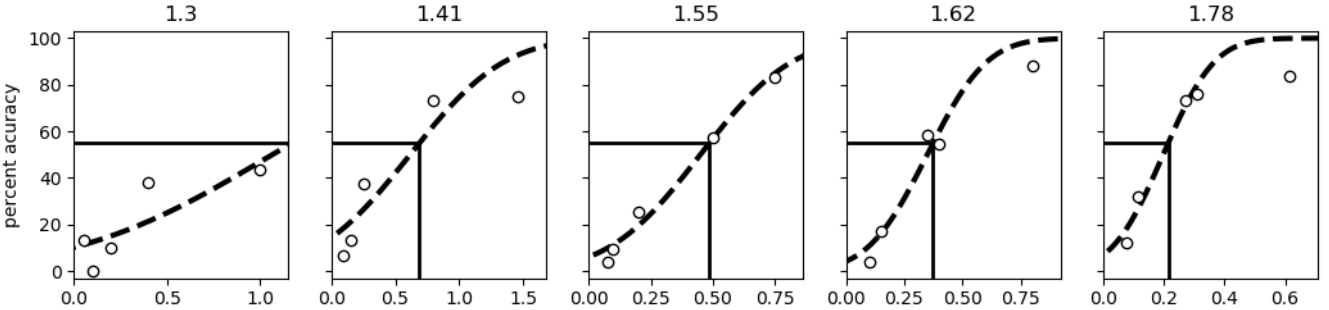

Sub 002

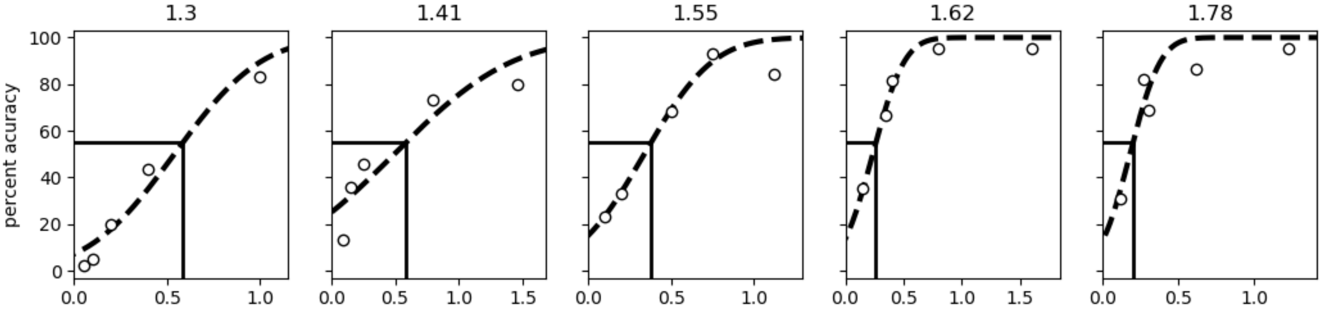

Sub 003

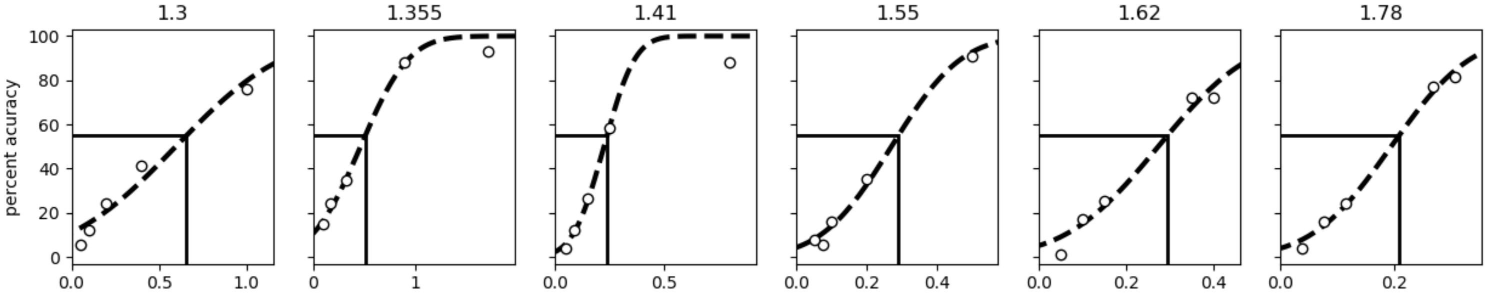

Sub 004

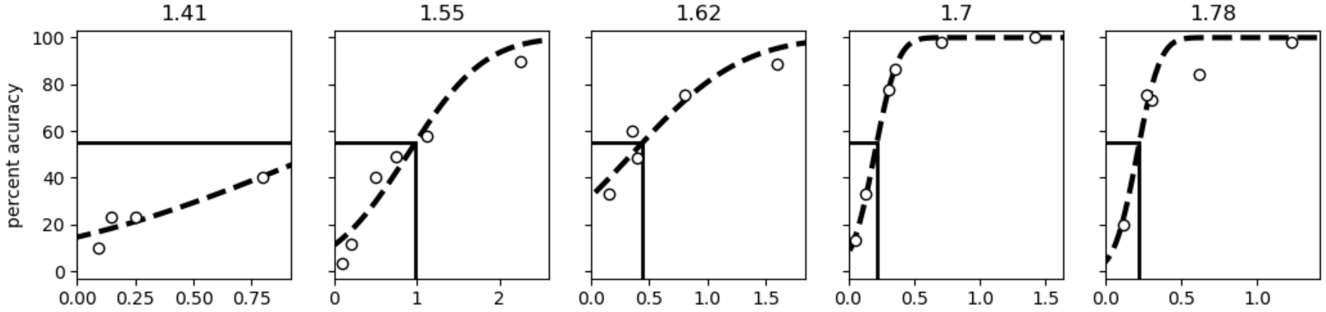

Sub 005

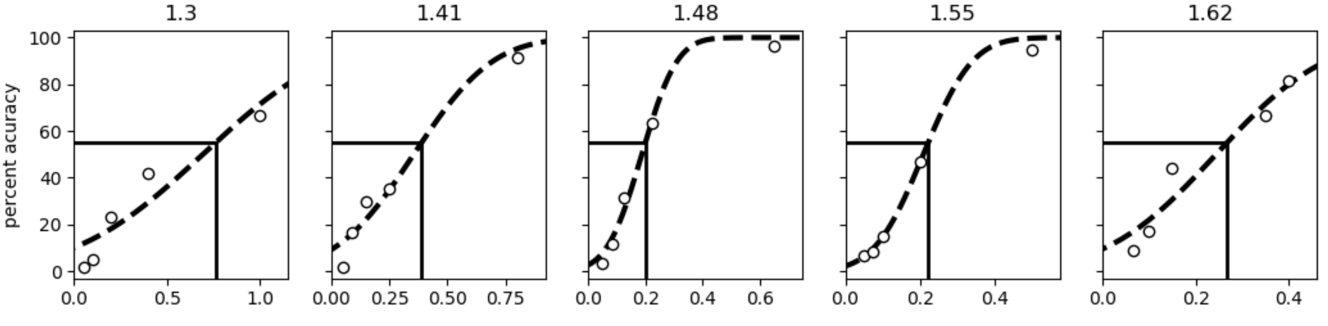

Sub 006

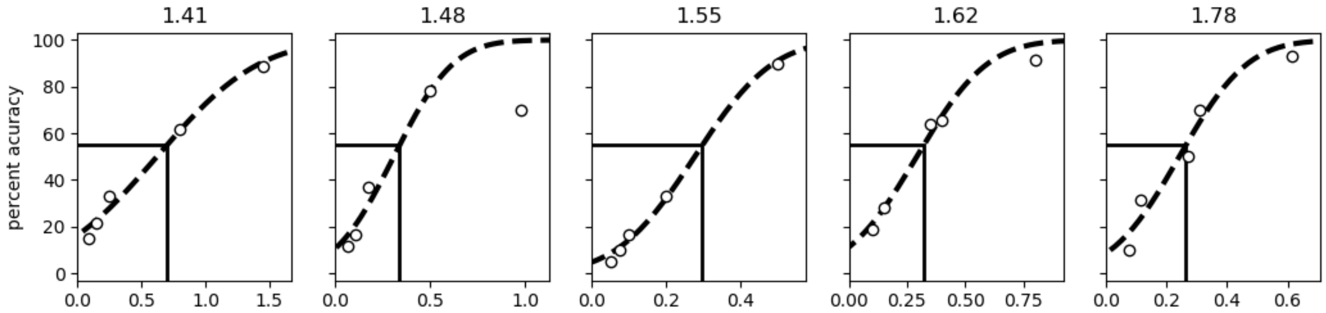

Sub 007

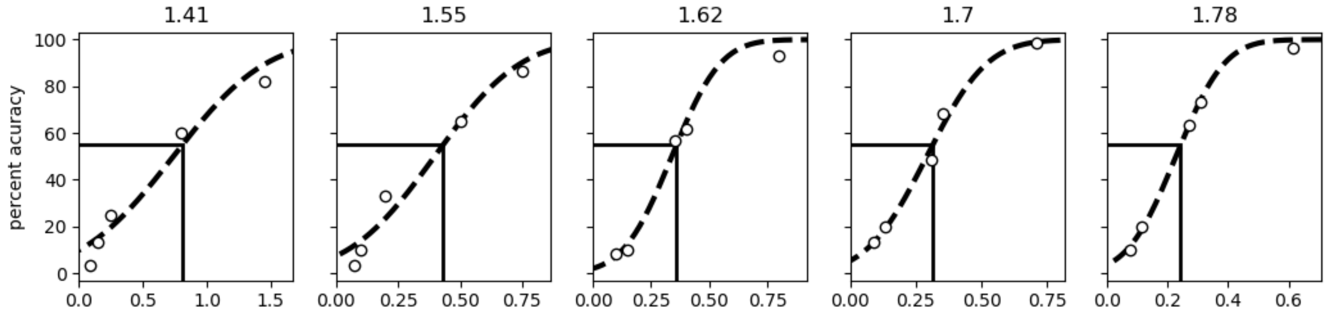

Sub 008

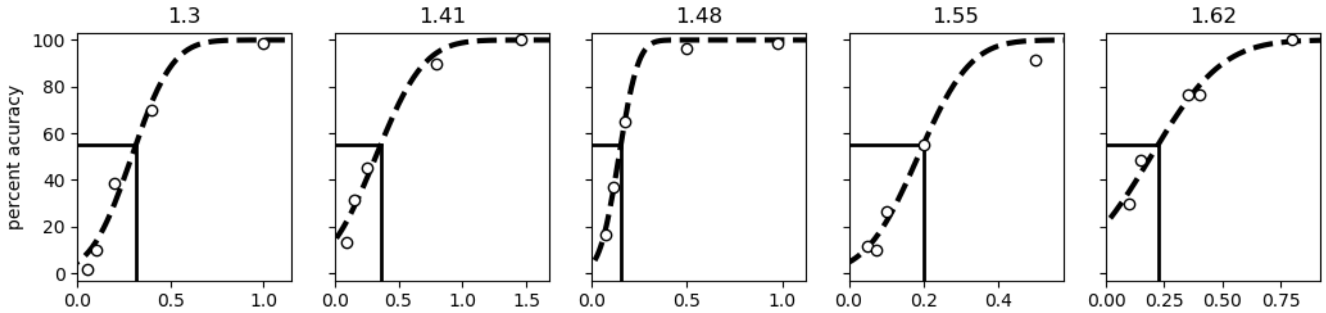

Sub 009

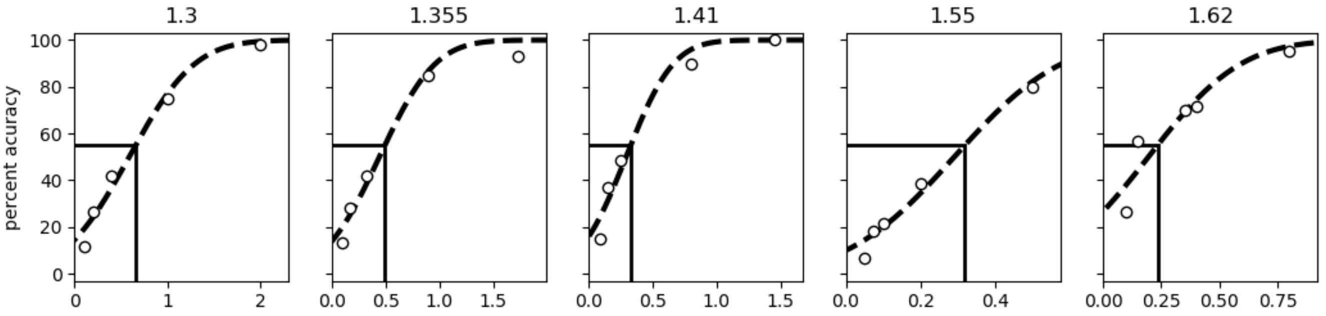

Sub 010

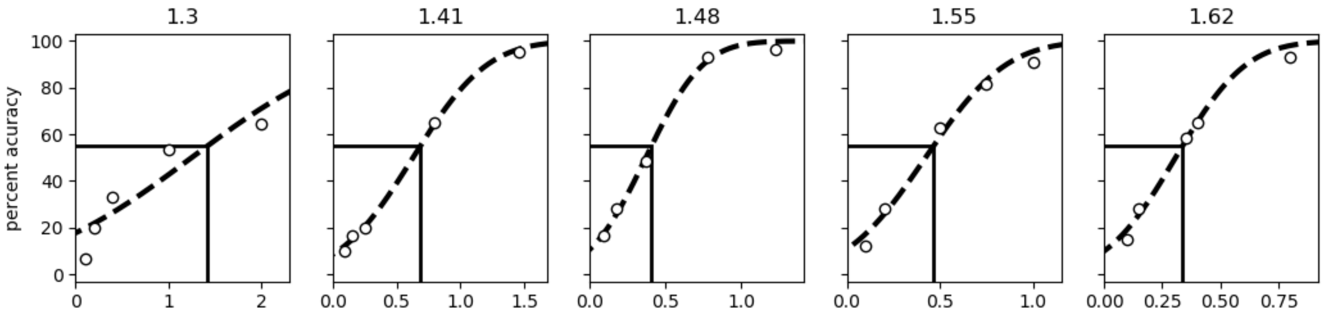

Sub 011

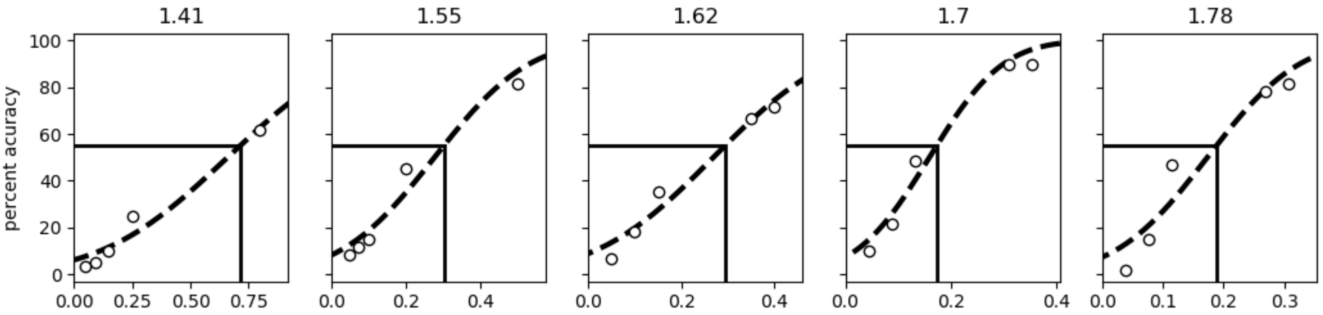

Sub 012

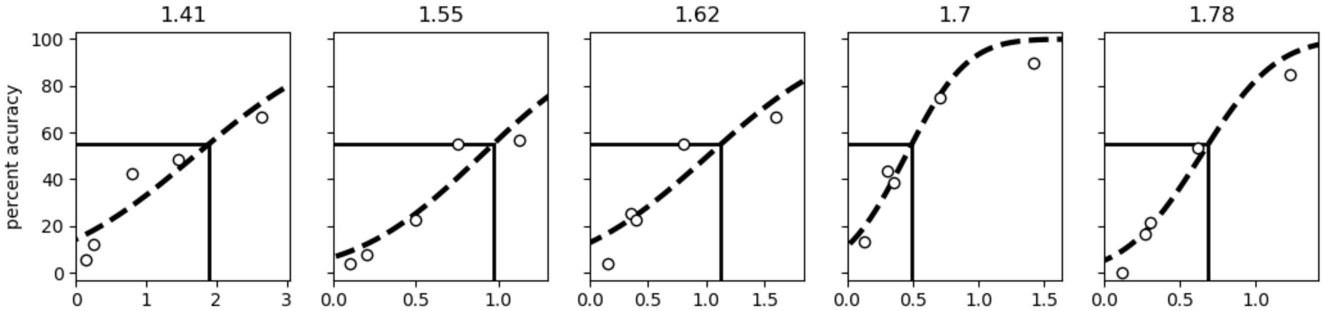

Sub 013

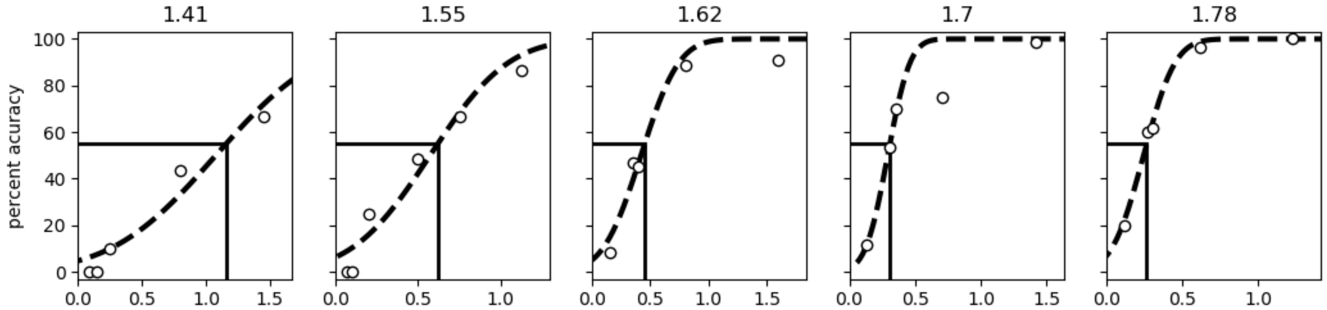

Sub 014

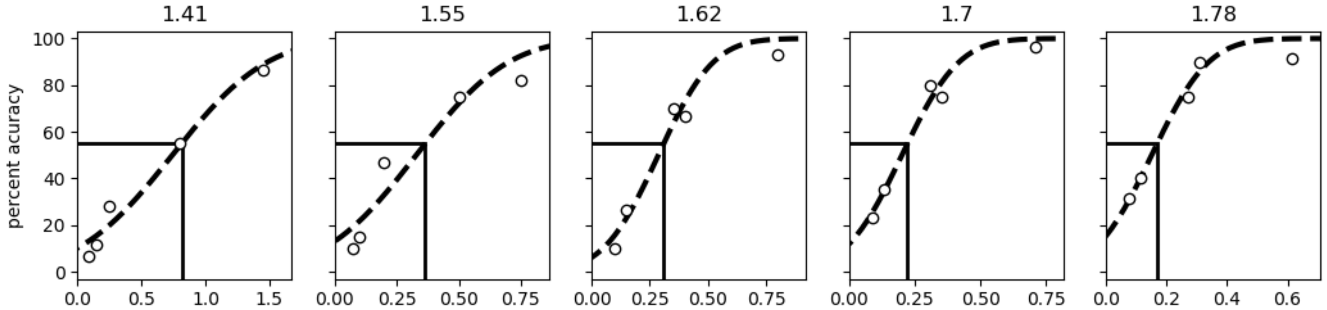

Sub 015

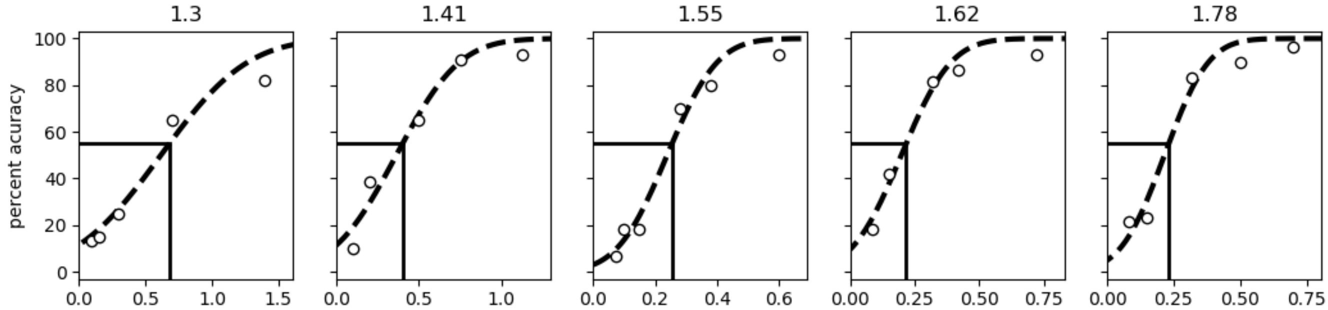

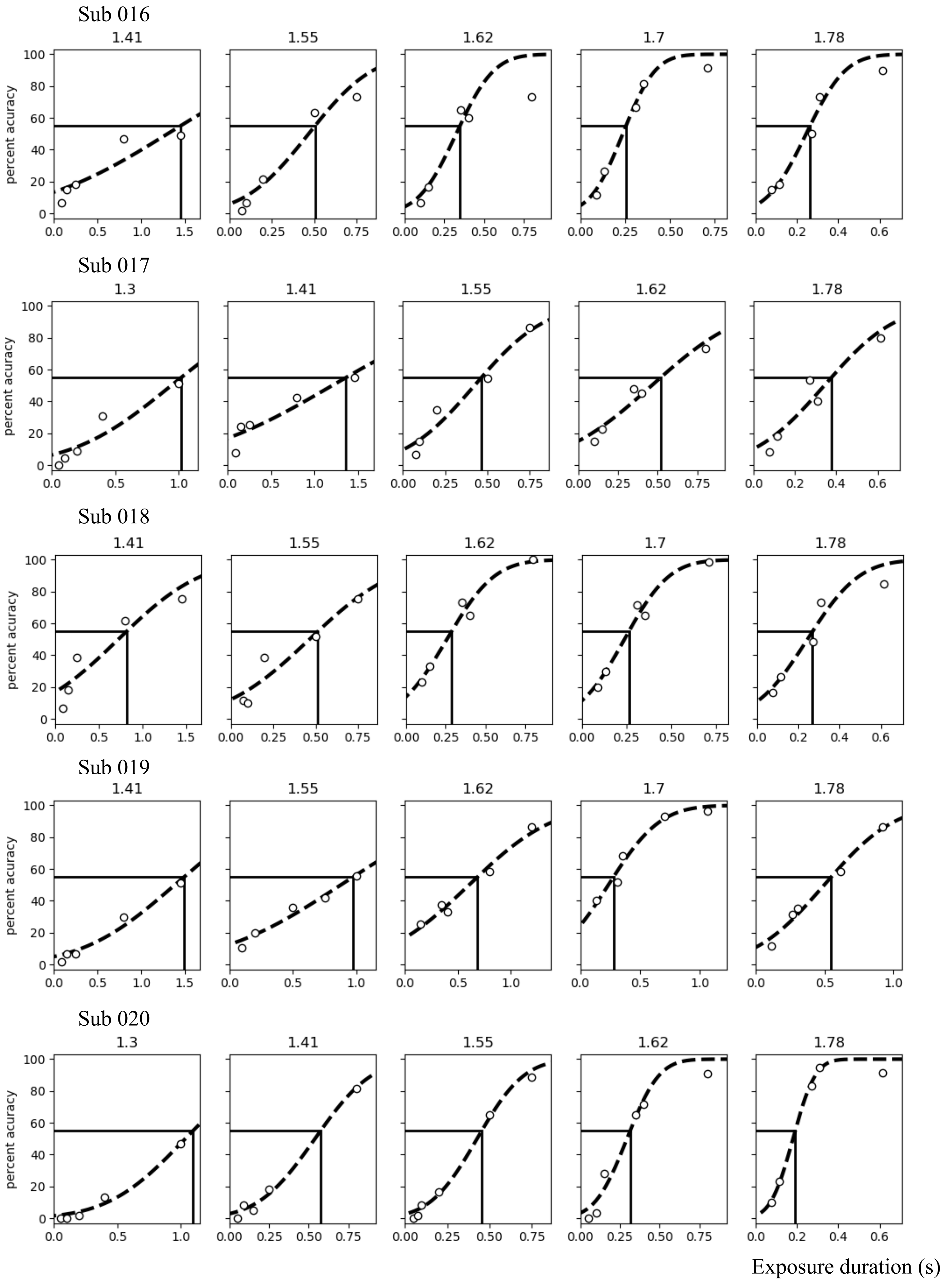
Sub 016
1.41
1.55
1.62
1.7
1.78
Sub 017
1.3
1.41
1.55
1.62
1.78
Sub 018
1.41
1.55
1.62
1.7
1.78
Sub 019
1.41
1.55
1.62
1.7
1.78
Sub 020
1.3
1.41
1.55
1.62
1.78
percent acuracy
Exposure duration (s)

2. *A-tDCS side effects*

The side effects of a-tDCS were assessed by asking participants if they experienced itching, pain, burning, tingling, or fatigue as a result of a-tDCS. The reported side effects were stronger in the active a-tDCS session compared with sham a-tDC (Wilcoxon matched-pairs signed rank test: $Z = -3.00$, $p < 0.01$, two-tailed, see Supplementary Table 1).

Supplementary Table 1. Participants' report of side effects due to a-tDCS at two stimulation sessions

Sub ID	Active a-tDCS					Sham a-tDCS				
	Itching	Pain	Burning	Tingling	Fatigue	Itching	Pain	Burning	Tingling	Fatigue
001	0	0	1	0	0	0	0	0	0	0
002	1	1	0	1	0	1	1	0	1	0
003	0	0	0	0	0	0	0	0	0	0
004	0	0	0	0	0	0	0	0	0	0
005	0	0	0	0	0	0	0	0	0	0
006	0	1	0	0	0	0	1	0	0	0
007	1	0	0	0	0	0	0	0	0	0
008	1	0	0	0	0	0	0	0	0	0
009	0	0	0	1	0	0	0	0	1	0
010	0	1	0	0	0	0	0	0	0	0
011	0	0	0	1	0	0	0	0	0	0
012	0	0	0	1	0	0	0	0	0	0
013	0	0	0	1	0	0	0	0	1	0
014	0	0	0	1	0	0	0	0	0	0
015	1	0	0	0	0	0	0	0	0	0
016	1	0	0	0	0	1	0	0	0	0
017	0	0	0	1	0	0	0	0	1	0
018	0	0	0	1	0	0	0	0	1	0
019	0	0	0	1	0	0	0	0	0	0
020	0	0	0	1	0	0	0	0	1	0
Proportion	0.25	0.15	0.05	0.50	0.00	0.10	0.10	0.00	0.30	0.00

1 indicates that subjects reported the presence of the given side effect;
0 indicates that subjects did not report the presence of the given side effect.
